# An Application of the Roentgen Ray to Infant Feeding

**Published:** 1915-02

**Authors:** 


### Abstracts and Selections.

### An Application of the Roentgen Ray to Infant Feeding.

Dr. Charles Hendee Smith presented this communication. He pointed out that the teaching of doctors and nurses for many years had been that the child must be kept at the breast or bottle for twenty minutes, must then be held in a perfectly horizontal position, and be put to sleep at once without change of position. Many mothers did not follow this plan, but allowed the child to take a shorter or longer time for nursing and then drew the following conclusions: (1) Regurgitation, colic, and indigestion depended not only on the food but on the manner in which it was given and the posture of the child between feedings. (2) The food must be of proper composition and proper amount. It should be given at as long intervals as possible, depending on the gastric capacity and the total amount of food required per day. (3) A feeding should not be taken too slowly, five to ten minutes being long enough and fifteen minutes being the maximum time. (4) The child should be held upright before feeding to get rid of any gas in the stomach. If air was swallowed in large quantities it was often necessary to interrupt the feeding one or more times, holding the child erect until the air had been expelled. Immediately after feeding the child should be held upright against the mother's shoulder and given the opportunity to eructate gas. (5) The child should be placed in bed preferably in the prone position with the head of the bed somewhat elevated. This routine might be followed with every child even though he did not regurgitate. Habitual tongue suckers had to be held up sometimes between feedings.—*Pediatrics.*

